# Transcriptional changes in multiple endocrine organs from lethal cases of COVID-19

**DOI:** 10.1007/s00109-023-02334-3

**Published:** 2023-05-29

**Authors:** Anello Marcello Poma, Diana Bonuccelli, Elisabetta Macerola, Sara Niballi, Alessio Basolo, Ferruccio Santini, Fulvio Basolo, Antonio Toniolo

**Affiliations:** 1 Department of Surgical, Medical, Molecular Pathology and Critical Area, University of Pisa, Via Roma, 67, 56126 Pisa, Italy; 2 Department of Forensic Medicine, Azienda USL Toscana Nordovest, Lucca, Italy; 3 Obesity and Lipodystrophy Center, Endocrinology Unit, University Hospital of Pisa, Pisa, Italy; 4 Global Virus Network, University of Insubria, Varese, Italy

**Keywords:** COVID-19, SARS-CoV-2, Leptin, Insulin, Endocrine tissues, Gene expression

## Abstract

**Abstract:**

Altered circulating hormone and metabolite levels have been reported during and post-COVID-19. Yet, studies of gene expression at the tissue level capable of identifying the causes of endocrine dysfunctions are lacking. Transcript levels of endocrine-specific genes were analyzed in five endocrine organs of lethal COVID-19 cases. Overall, 116 autoptic specimens from 77 individuals (50 COVID-19 cases and 27 uninfected controls) were included. Samples were tested for the SARS-CoV-2 genome. The adrenals, pancreas, ovary, thyroid, and white adipose tissue (WAT) were investigated. Transcript levels of 42 endocrine-specific and 3 interferon-stimulated genes (ISGs) were measured and compared between COVID-19 cases (virus-positive and virus-negative in each tissue) and uninfected controls. ISG transcript levels were enhanced in SARS-CoV-2-positive tissues. Endocrine-specific genes (e.g., *HSD3B2*, *INS*, *IAPP*, *TSHR*, *FOXE1*, *LEP*, and *CRYGD*) were deregulated in COVID-19 cases in an organ-specific manner. Transcription of organ-specific genes was suppressed in virus-positive specimens of the ovary, pancreas, and thyroid but enhanced in the adrenals. In WAT of COVID-19 cases, transcription of ISGs and leptin was enhanced independently of virus detection in tissue. Though vaccination and prior infection have a protective role against acute and long-term effects of COVID-19, clinicians must be aware that endocrine manifestations can derive from virus-induced and/or stress-induced transcriptional changes of individual endocrine genes.

**Key messages:**

• SARS-CoV-2 can infect adipose tissue, adrenals, ovary, pancreas and thyroid.

• Infection of endocrine organs induces interferon response.

• Interferon response is observed in adipose tissue independently of virus presence.

• Endocrine-specific genes are deregulated in an organ-specific manner in COVID-19.

• Transcription of crucial genes such as *INS*, *TSHR* and *LEP* is altered in COVID-19.

**Supplementary Information:**

The online version contains supplementary material available at 10.1007/s00109-023-02334-3.

## Introduction

The severe acute respiratory syndrome coronavirus 2 (SARS-CoV-2) causes COVID-19, a disease presenting with a spectrum of manifestations that range from asymptomatic infection to severe pneumonia followed by multiorgan failure [1]. The reasons why severe forms of COVID-19 may occur are controversial, but evidence suggests that defects of the interferon (IFN) response and/or autoantibodies to IFNs are of prime importance [2]. Generally, it is agreed that mild COVID-19 forms associate with a prompt and robust type I IFN response that limits virus replication and spreading. This response is typical of young people. Severe forms of COVID-19 are instead associated with a delayed type I IFN response that allows an extensive replication of the virus and its abundant spread within the body, including the endocrine organs [3–7].

Though the pathogenesis is unclear, clinical studies show that multiple endocrine functions may be altered in COVID-19. Obesity is an important risk factor for hospitalization and mortality [8]. In fact, SARS-CoV-2 replicates in macrophages and adipocytes [6, 9], and the adipose tissue is an important virus reservoir. Obese subjects have a systemic pro-inflammatory state with abnormal production of adipokines, hyperglycemia, and metabolic disorders. In COVID-19, high glucose levels associate with a remarkable increase of acute respiratory distress syndrome that leads to mechanical ventilation and high mortality [10]. Several mechanisms contribute to hyperglycemia, including insulin resistance associated with systemic inflammation, administration of glucocorticoids, and the direct infection of pancreatic islets [11]. Though cases of new-onset diabetes following COVID-19 have been reported [12], it is debated whether SARS-CoV-2 may infect human pancreatic cells. van der Heide and colleagues found that productive infection is strictly dependent on the expression of the entry receptors and targets practically all pancreatic cell types. However, infection is little cytopathic and promotes only modest cellular perturbations [13]. Other authors observed that SARS-CoV-2 selectively infects pancreatic beta cells in vitro attenuating insulin secretion and inducing apoptosis [14].

Glucocorticoids are produced by adrenal glands and are essential to balance the immune response [15]. In critically ill COVID-19 patients, reduced levels of cortisol and adrenocorticotrophic hormone (ACTH) are observed [16]. Cases of central adrenal insufficiency have been reported [17], and, at autopsy, the genome and antigens of SARS-CoV-2 have been detected in adrenals [18]. However, pathological changes of adrenals in COVID-19 are minimal [18, 19], and it is debated whether adrenal insufficiency is primary or secondary to impairment of the hypothalamic–pituitary–adrenal (HPA) axis. In this context, COVID-19-associated transcriptional changes of pituitary genes could play a role [5].

Similarly, impairment of the hypothalamic-pituitary-thyroid (HPT) axis has been observed and might contribute to thyroid dysfunction. Though many patients are euthyroid, some of them—especially those admitted to the intensive therapy unit—manifest clinical hypothyroidism with reduced levels of both thyroid-stimulating hormone (TSH) and free thyroxine (T4) [20]. In addition, Lania et al. [21] reported thyrotoxicosis with a risk of atrial fibrillation and thromboembolic events in about 20% of patients hospitalized with severe COVID-19.

Finally, reduced levels of estradiol and dihydrotestosterone have been found in female and male COVID-19 patients with sex-specific patterns of hypogonadism [22]. Very low testosterone levels associate with severe forms of COVID-19 in male patients, while in females, both the anti-Müllerian hormone and estradiol showed a negative correlation with severity of infection [23]. SARS-CoV-2 may infect ovaries [24], and, during recovery from COVID-19, a temporary decrease of menstrual volume or cycle prolongation was observed in about 20% of women of childbearing age[25].

To sum up, numerous studies addressed hormonal changes in COVID-19, but studies of virus-associated changes in endocrine organs remain scarce and limited to histopathological observations. In lethal COVID-19 cases, we evaluated mRNA transcript levels of tissue-specific genes in five endocrine organs: adrenal gland, pancreas, ovary, thyroid, and the abdominal subcutaneous white adipose tissue (WAT). Levels of mRNA transcripts have been compared to those of a matched control group of subjects dying abruptly of non-infectious causes. Results indicate that severe forms of COVID-19 are characterized by the activation of type I IFN pathways and by significant alterations of organ-specific endocrine transcripts that may lead to clinical dysfunctions.

## Materials and methods

### Investigated cases and controls

One hundred sixteen autopsy specimens from 77 individuals were included in the study. Fifty of them died of COVID-19, while 27 subjects dying abruptly of non-infectious causes (trauma, sudden cardiac death) served as controls. Autopsies have been performed in a Biosafety Level 3 facility at the Unit of Forensic Medicine (Azienda USL Toscana Nord Ovest, Lucca, Italy) serving four tertiary care Hospitals: Pisa, Lucca, Livorno, and Massa-Carrara. In COVID-19 cases (but not in controls), two post-mortem lung biopsies tested positive for the SARS-CoV-2 genome using real-time reverse transcription polymerase chain reaction (RT-PCR). The pathology report of COVID-19 cases confirmed the infection as a primary cause of death for respiratory failure, at times accompanied by multiorgan failure. At histology, the lung parenchyma showed extensive alveolar damage, involvement of endothelial/interstitial cells, interstitial and alveolar edema, hemorrhages, microthrombi, hyaline membranes, and mononuclear cell infiltrations with macrophages. No significant histological changes were observed in the lungs of uninfected controls.

Five different endocrine organs were studied: adrenal gland, pancreas, ovary, thyroid, and WAT. Some cases overlap with cases that had been investigated in previous reports of our group [3–6].

### RT-PCR and nCounter assay

For each case, four to six 10-μm-thick formalin-fixed paraffin-embedded (FFPE) sections were used for RNA isolation using the RNeasy FFPE kit (Qiagen, Hilden, Germany). RNA quality and quantity were assessed using an Xpose spectrophotometer (Trinean, Gentbrugge, Belgium). Total RNA (250 ng) was utilized for detecting the viral genome using the one-step Easy SARS-CoV-2 WE RT-PCR kit (Diatech Pharmacogenetics, Jesi, Italy) as described [5]. Briefly, the assay has a limit of detection of 5 target copies per reaction. Two virus targets are tested, namely, the nucleocapsid (N) and the RNA-dependent RNA polymerase (RdRp) genes. A sample was considered positive when at least one of the targets was amplified at Ct values below those indicated by the manufacturer (i.e., 36th Ct for N and 38th Ct for RdRp).

Gene expression levels were measured by the nCounter system (NanoString Technologies, Seattle, WA, USA) using a custom 55-gene panel that included 10 housekeeping genes used as reference (i.e., *ABCF1*, *ALAS1*, *GUSB*, *MRPS7*, *NMT1*, *NRDE2*, *OAZ1*, *PGK1*, *SDHA*, and *STK11IP*), 3 IFN-stimulated genes (ISGs; i.e., *IFI44*, *OAS1*, and *RSAD2*), and 42 genes that were specifically expressed in the investigated endocrine organs. Endocrine-specific genes had been selected based on representing the top tissue-enriched or group-enriched genes in the Human Protein Atlas (https://www.proteinatlas.org). As shown in Supplementary Table S1, the following endocrine-specific genes were studied: adrenal gland (*n* = 8), *CCN3*, *CYP11B1*, *CYP11B2*, *CYP17A1*, *CYP21A2*, *GML*, *HSD3B2*, and *KCNK2*; ovary (*n* = 6), *CRYGD*, *HTR1A*, *KLHDC8A*, *LEFTY2*, *NXPH2*, and *WFIKKN2*; pancreas (*n* = 11), *CPA1*, *CTRB2*, *GAD1*, *GAD2*, *GCG*, *IAPP*, *INS*, *PNLIPRP1*, *PTPRN*, *SLC30A8*, and *SST*; thyroid (*n* = 12), *BMP8A*, *FOXE1*, *GOLGA8Q*, *ID4*, *IYD*, *PKHD1L1*, *SLC26A4*, *SLC26A7*, *TG*, *TPO*, *TSHR*, and *ZNF804B*; and WAT (*n* = 5), *AQP7B*, *LEP*, *OR52N5*, *TM4SF19*, and *TRAG1*. For the nCounter assay, total RNA (175 ng) was hybridized with probes at 65 °C for 21 h.

### Data analysis and statistics

Raw mRNA transcript counts were normalized following the procedures of the advanced analysis module of the nSolver software v.4.0 (NanoString Technologies). Genes with an expression level above the mean plus two standard deviations of negative control probes in a proportion equal to the size of the smallest group were used for further analyses. Normalized counts were log2-transformed for downstream analyses. Principal component analysis (PCA) was performed using the filtered genes and following the procedures of PCAtools Bioconductor package v.2.8.0. Unsupervised clustering was carried out using heatmap3 R package v.1.1.9 and setting Euclidean and Ward as distance and clustering method, respectively. Differentially expressed genes (DEG) were computed using the best-fitting model among negative binomial, simplified negative binomial, and log-linear. Age, sex, and body mass index (BMI) were used in the model as confounders. Control cases were used as a baseline, and two comparisons were made for each tissue type: COVID-19 cases that were virus-positive in the endocrine tissue vs*.* controls; COVID-19 cases virus-negative in the endocrine tissue vs. controls. The Benjamini–Hochberg method was used to adjust *P* values. A false discovery rate (FDR) of 0.25 was considered significant. The analyses were performed in R environment v.4.1.2 (https://www.r-project.org/, last accessed March 31, 2023), unless otherwise specified.

## Results

**Fig. 1:**
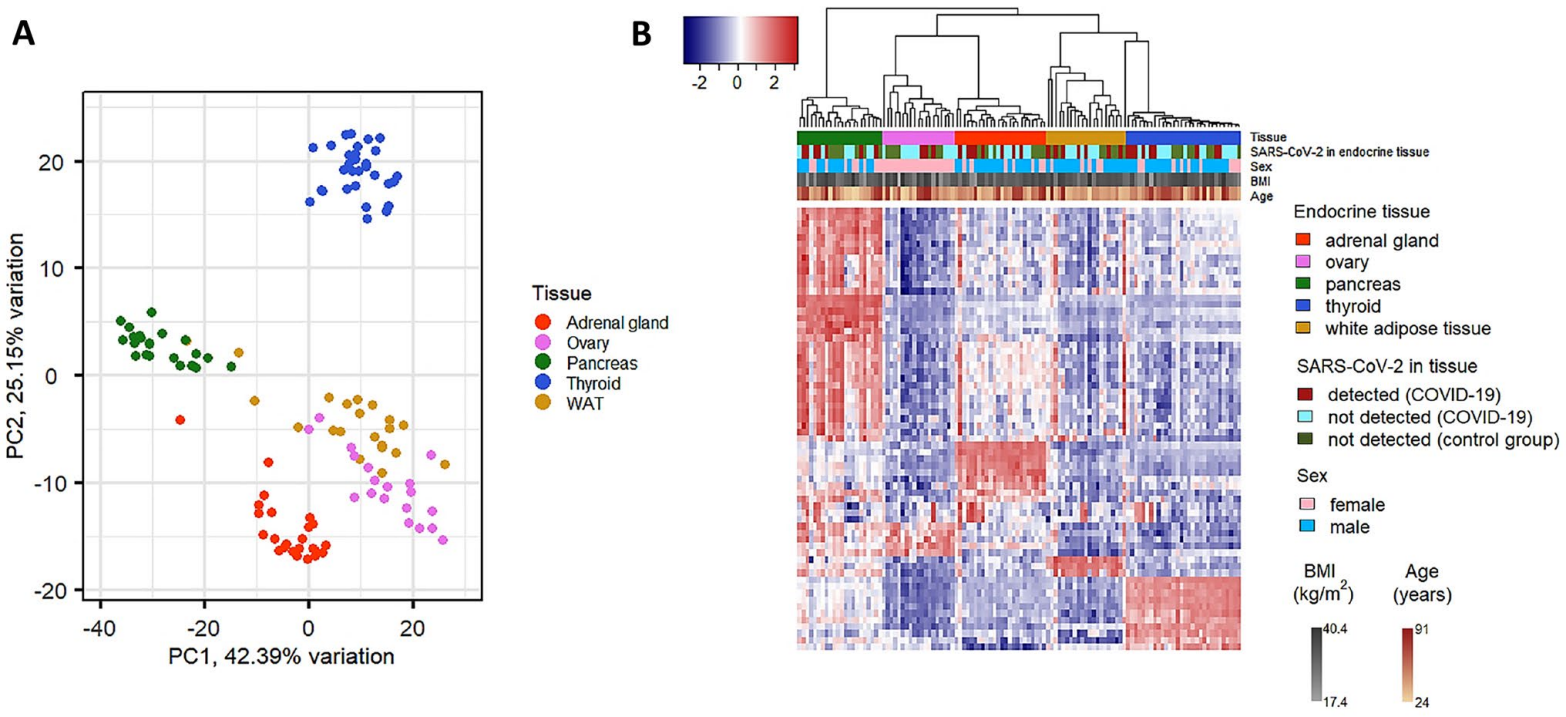
Organ-specific expression of endocrine genes. Transcript levels of endocrine genes were expressed in an organ-specific manner. **A** Principal component analysis was performed using the entire set of endocrine genes. Unadjusted normalized log2 counts were used. Principal components 1 and 2 that account for the majority of variation (67%) were plotted, and a neat separation of samples according to organ type can be observed. Results were confirmed by unsupervised clustering (**B**). Samples were clustered based on Euclidean distance and Ward’s minimum variance method. Five different clusters specific for each organ type were produced. No effect of age, sex, BMI, and detection/absence of SARS-CoV-2 could be observed at this level

**Fig. 2:**
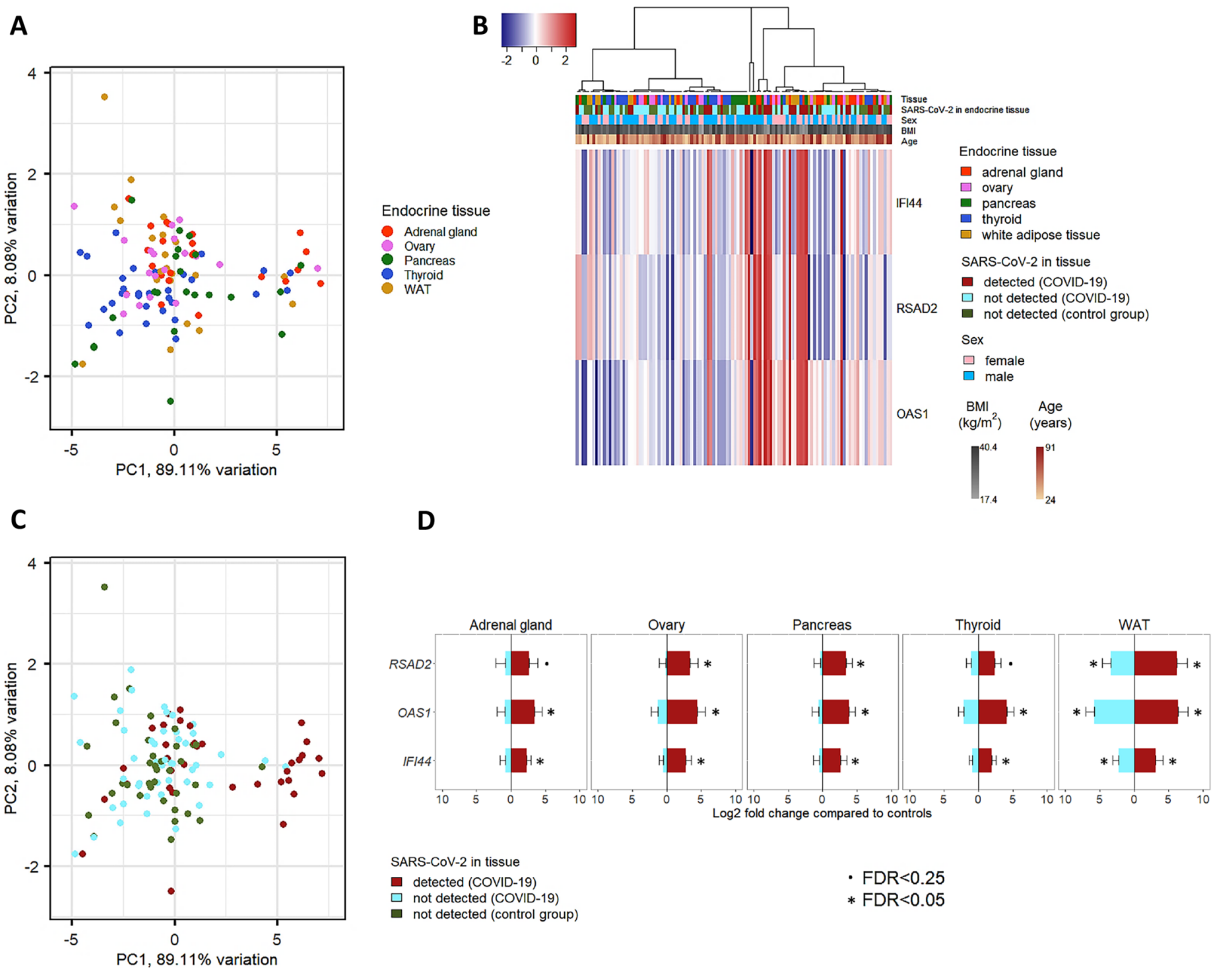
Expression of IFN-stimulated genes (ISGs). Principal component analysis (PCA) and hierarchical clustering were performed using the 3 ISG (i.e., *IFI44*, *OAS1*, and *RSAD2*) unadjusted counts. Clustering was carried out using Euclidean distance and Ward’s method. No effect of organ type was observed both on PCA (**A**) and by hierarchical clustering (**B**). A cluster enriched in specimens positive for the SARS-CoV-2 genome can be observed. This is confirmed on PCA (**C**), where a group of SARS-CoV-2-positive samples (in red) shows a peculiar expression pattern. **D** shows the log2 fold change of ISGs (i.e., *RSAD2*, *OAS1*, and *IFI44*) in virus-positive (red) and virus-negative (cyan) COVID-19 samples compared to the baseline of controls. In all organ types, ISGs are significantly upregulated only when the virus could be detected in the tissue. The only exception is the WAT, where enhanced transcription of ISGs is observed also in virus-negative COVID-19 specimens

Normalized gene transcription levels were used to evaluate the organ specificity of endocrine-related genes. As shown in Fig. 1, using the entire set of genes, the organ type was the major determinant of variation. Organ-specific clusters were observed using both principal component analysis (PCA) and hierarchical clustering. The analyses did not detect any significant effects for other variables (i.e., virus detection into the tissue, sex, age, and body mass index (BMI)). Figure 2 shows that transcription of IFN-stimulated genes (ISGs, i.e., *OAS1*, *RSAD2*, *IFI44*) was independent on the type of endocrine organ (Fig. 2A, B), and—as expected—transcription of ISGs was activated in tissues in which SARS-CoV-2 was detected (Fig. 2B, C). Figure 2D further demonstrates that upregulated transcription of ISGs occurred in virus-positive but not in virus-negative endocrine organs. One notable exception was WAT (Fig. 2D) that showed activated transcription of ISGs independently of virus detection in the tissue.

Below is the analysis of transcript levels of endocrine-specific genes per individual organ. Clinical data including COVID-19 treatment and previous comorbidities for all cohorts per each organ are reported in Supplementary Tables S2-6.

### Adrenal gland

**Fig. 3:**
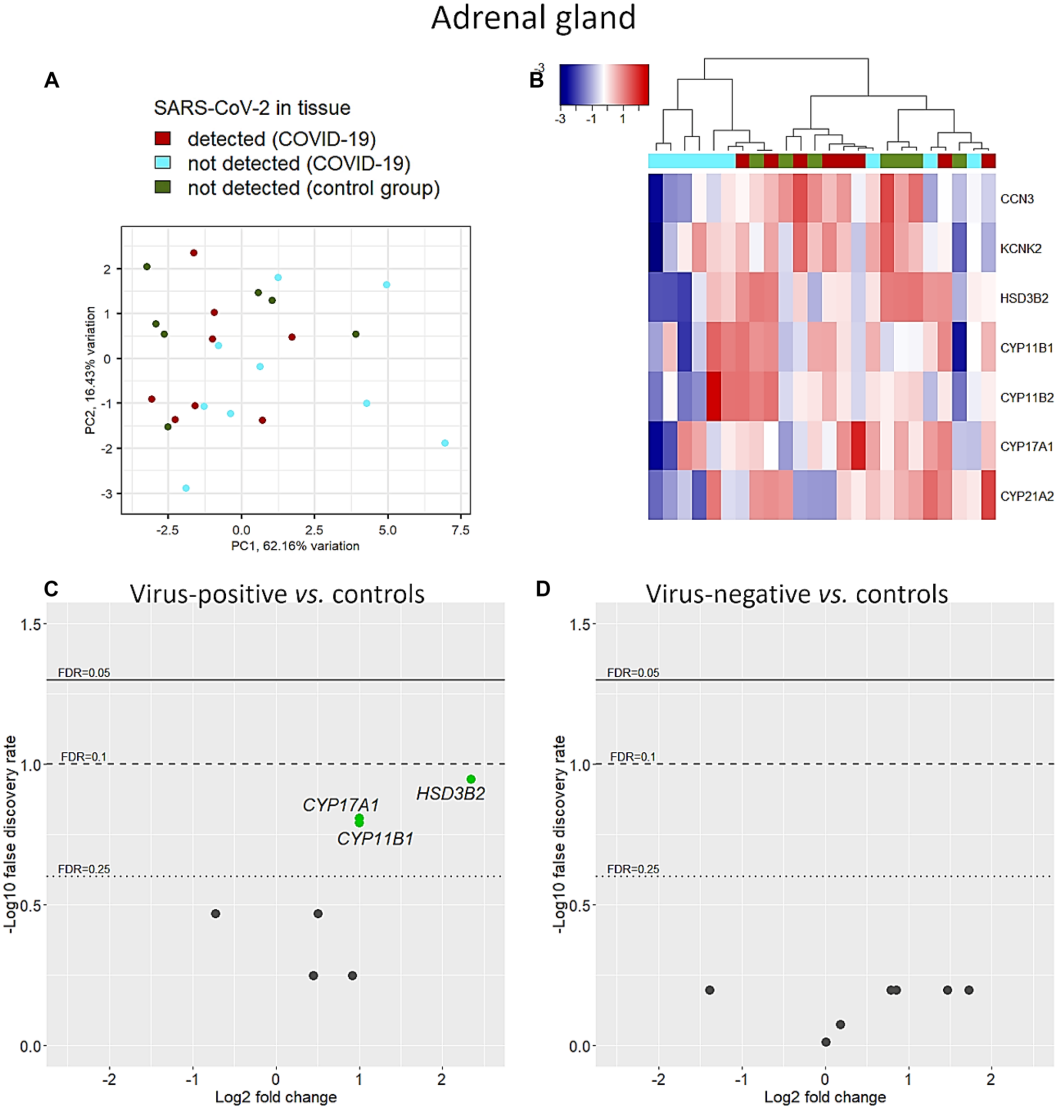
Adrenal-specific genes. Unadjusted transcript levels of adrenal genes did not produce specific clusters on principal component analysis (**A**) nor on clustering using Euclidean distance and Ward’s method (**B**). However, after adjusting for confounders (i.e. sex, age, and BMI), 3 genes (i.e., *HSD3B2*, *CYP17A1*, and *CYP11B1*) were significantly upregulated in SARS-CoV-2-positive adrenals (**C**), while no differences were observed in virus-negative COVID-19 specimens compared to controls (**D**)

Seven of 8 adrenal-specific genes passed the quality checks. Only *GML* was filtered out due to low counts. PCA and hierarchical clustering showed slightly different patterns of transcription without a clear distinction of COVID-19 from uninfected control cases (Fig. 3A, B). Deregulated endocrine-specific genes were observed only in virus-positive adrenal specimens, while no significant transcriptional changes were detected in virus-negative adrenals of COVID-19 cases (Fig. 3C, D). Three genes were upregulated in virus-positive adrenal tissues (Fig. 3C): hydroxy-delta-steroid dehydrogenase, 3 beta- and steroid delta-isomerase 2 (*HSD3B2*, FC = 2.34, FDR = 0.11), and cytochrome P450 family 17 subfamily A member 1 (*CYP17A1*, FC = 1, FDR = 0.15) and cytochrome P450 family 11 subfamily B member 1 (*CYP11B1*, FC = 1, FDR = 0.16).

### Ovary

**Fig. 4:**
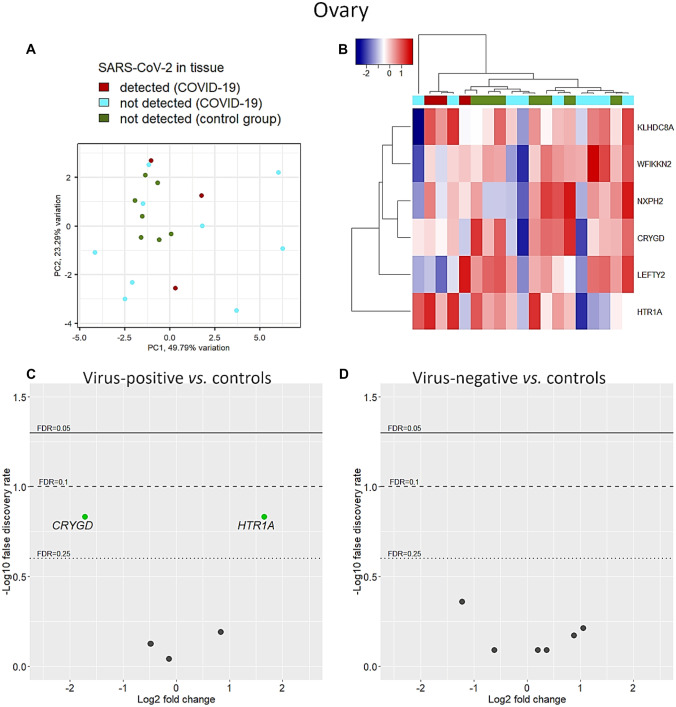
Ovary-specific genes. Principal component analysis (**A**) and clustering with Euclidean distance and Ward’s method (**B**) were carried out using unadjusted counts. Ovary-specific genes were heterogeneously expressed in COVID-19 samples (**A**, **B**). However, after adjusting for age, sex, and BMI, in virus-positive tissues, *CRYGD* transcripts were significantly suppressed, while *HTR1A* transcript levels were enhanced (**C**). The SARS-CoV-2-negative COVID-19 cohort did not show deregulations of any gene (**D**)

Six ovary-specific genes were considered for analysis. While uninfected controls showed a clustered transcription pattern, the expression profile was highly variable in COVID-19 cases (Fig. 4A, B). Interestingly, ovaries negative for SARS-CoV-2 showed no deregulation of endocrine-specific genes, whereas virus-positive ovaries showed significant deregulation of two genes (Fig. 4C, D). Transcription of crystallin gamma D (*CRYGD*) was downregulated (FC = −1.71, FDR = 0.15) while that of 5-hydroxytryptamine receptor 1A (*HTR1A*) was enhanced (FC = 1.66, FDR = 0.15). Consistent with our findings, exposure to type I IFN leads to enhanced transcription of *HTR1A* in the bovine endometrium [26].

### Pancreas

**Fig. 5:**
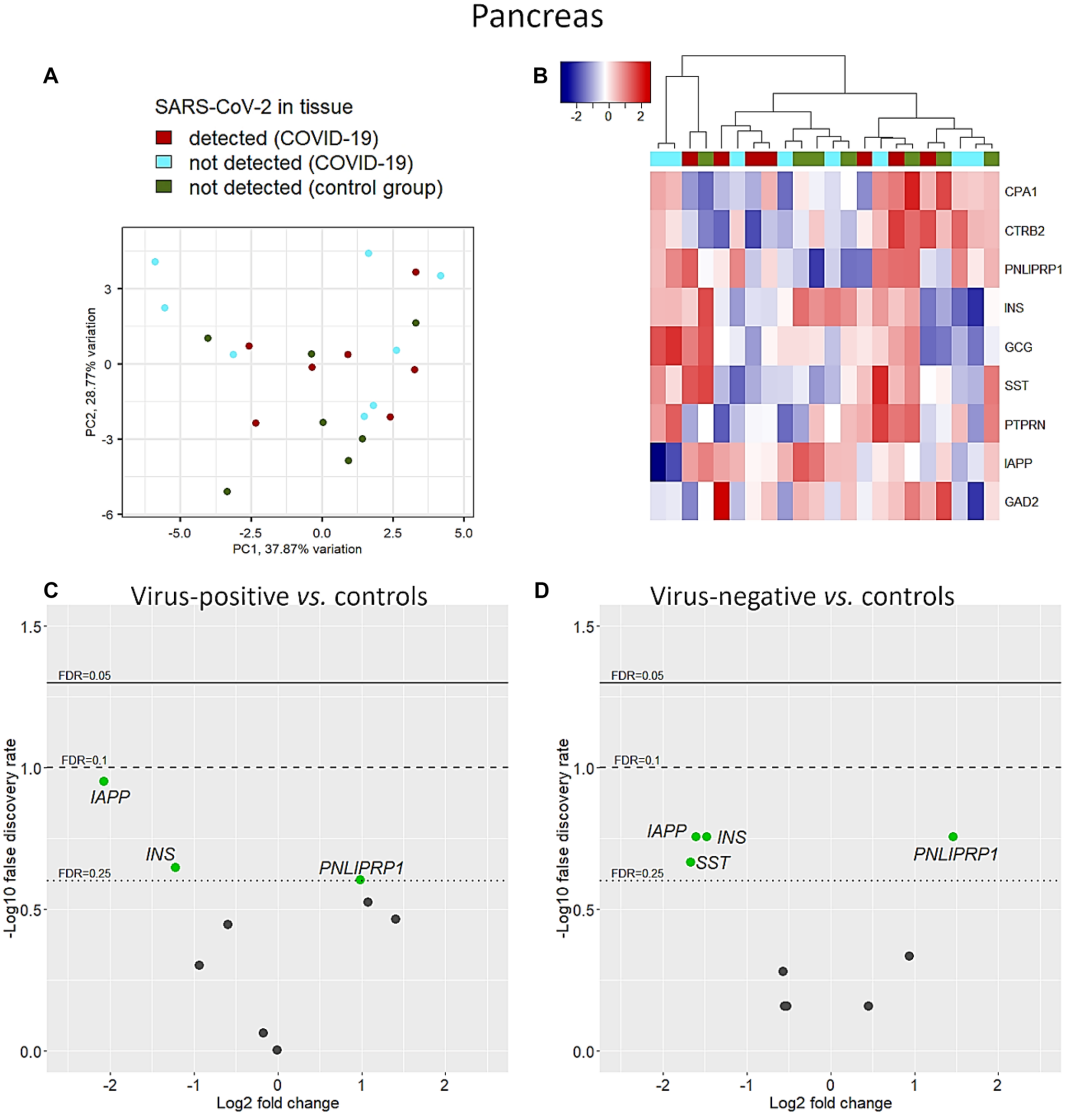
Pancreas-specific genes. Like in the majority of organs, unadjusted level of specific transcripts did not clearly separate pancreatic specimens according to COVID-19 or virus positivity on principal component analysis (**A**) nor on clustering with Euclidean distance and Ward’s method (**B**). Differential expression analysis using age, sex, and BMI as confounders, however, produced 2 downregulated (i.e., *IAPP* and *INS*) and 1 upregulated (*PNLIPRP1*) genes in virus-infected tissues (**C**) and in COVID-19 specimens in which the virus was not detected. Notably, somatostatin (*SST*) transcript levels were downregulated in the virus-negative COVID-19 group (**D**)

After normalization, 2 genes (i.e., *GAD1* and *SLC30A8*) were filtered out because of low counts, while 9 pancreas-specific genes were considered for downstream analysis. PCA showed a degree of separation between COVID-19 and uninfected control cases on principal component 2, but some overlapping remained (Fig. 5A). Similarly, hierarchical clustering did not produce specific clustering in relation to COVID-19 diagnosis nor to virus-positivity within the organ (Fig. 5B). However, when adjusting for confounders (i.e., age, sex, BMI), transcription of 3 genes was deregulated in COVID-19 cases independently of SARS-CoV-2 detection in the tissue (Fig. 5C, D). Transcription of the pancreatic lipase related protein 1 (*PNLIPRP1*) was enhanced in COVID-19 cases vs. controls (FC = 0.98, FDR = 0.25 in virus-positive; FC = 1.46, FDR = 0.17 in virus-negative tissues). With regard to endocrine-specific genes, COVID-19 cases were characterized by suppressed levels of hormonal transcripts in both virus-positive and virus-negative cases compared to controls (insulin (*INS*) FC = −1.22, FDR = 0.22 and FC = −1.47, FDR = 0.17 respectively; islet amyloid polypeptide (*IAPP*) FC = −2.07, FDR = 0.11 in virus-positive and FC = −1.67, FDR = 0.21 in virus-negative cases). Of note, transcripts of the somatostatin precursor gene (*SST*) were downregulated only in virus-negative COVID-19 cases (FC = −1.6, FDR = 0.17) but did not changed in virus-positive specimens.

### Thyroid

**Fig. 6:**
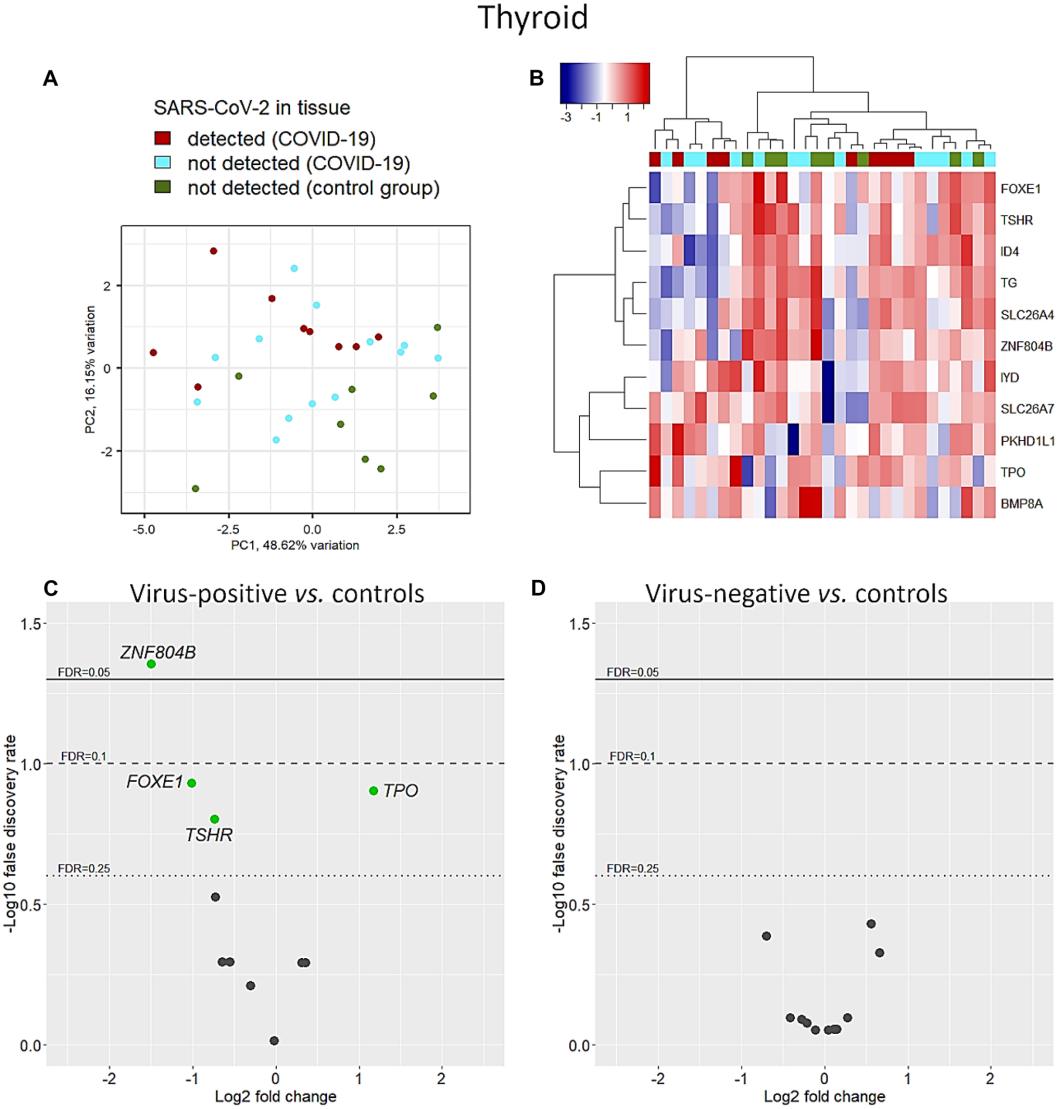
Thyroid-specific genes. Unlike other organs, distinct patterns of expression between COVID-19 and controls were observed on principal component analysis in thyroid specimens using unadjusted thyroid-specific transcript levels (**A**). Similarly, clustering with Euclidean distance and Ward’s method produced distinct clusters, though not completely specific (**B**). After adjusting for age, sex, and BMI, in virus-positive samples, *TPO* transcription was enhanced compared to controls, while mRNA transcript levels of *ZNF804B*, *FOXE1*, and *TSHR* were downregulated (**C**). No gene deregulation was observed in COVID-19 cases that were negative for SARS-CoV-2 in tissue (**D**)

Eleven out of 12 genes passed the quality check and were included for further analyses. *GOLGA8Q* was filtered out. PCA showed different patterns of transcription between COVID-19 cases and controls. Changes were particularly evident in virus-positive specimens (Fig. 6A). Differences failed to emerge in the clustering analysis, in which only a subgroup of COVID-19 cases had a distinct expression profile (Fig. 6B). In virus-positive specimens, the expression of 3 genes was downregulated (Fig. 6C): zinc finger protein 804B (*ZNF804B*, FC = −1.49, FDR = 0.04), forkhead box E1 (*FOXE1*, also called thyroid transcription factor 2, FC = −1.01, FDR = 0.12), and thyroid-stimulating hormone receptor, the main autoantigen in Graves’ disease (*TSHR*, FC = −0.73, FDR = 0.16). Interestingly, virus-positive specimens showed enhanced transcription of thyroid peroxidase (TPO), a major autoantigen in autoimmune thyroid disorders (FC = 1.18, FDR = 0.12). Finally, as compared to controls, gene transcript levels were not significantly altered in virus-negative cases of COVID-19 (Fig. 6D).

### White adipose tissue (WAT)

**Fig. 7:**
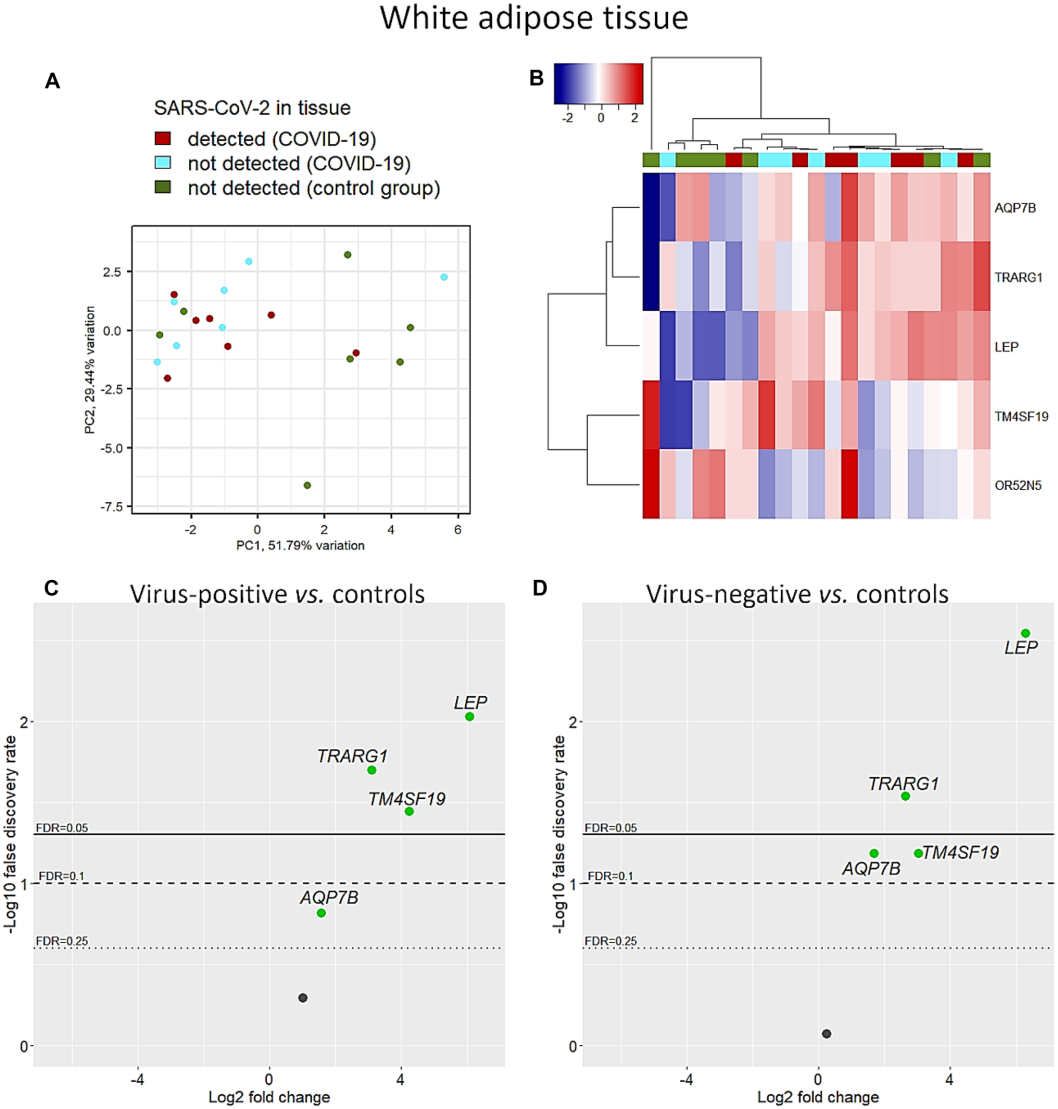
WAT-specific genes. Some differences between COVID-19 and control cases were observed using unadjusted gene expression levels both by principal component analysis (**A**) and hierarchical clustering with Euclidean distance and Ward’s method (**B**). After adjusting for confounders (i.e., age, sex, and BMI), both virus-positive (**C**) and virus-negative (**D**) COVID-19 cases showed upregulation of 4 genes compared to the control group, especially the leptin (*LEP*) gene, accompanied by the *TRARG1*, *TM4SF19*, and *AQP7B* genes

Five WAT-specific genes passed quality checks. With a few exceptions, unsupervised analyses showed a separation of COVID-19 cases from controls (Fig. 7A, B). Four of 5 WAT-specific genes were significantly upregulated in COVID-19 independently of virus detection in the tissue (Fig. 7C, D). The leptin (*LEP*) gene showed the highest upregulation level both in virus-positive and in virus-negative COVID-19 cases (FC = 6.07, FDR = 0.009 and FC = 6.28, FDR = 0.003, respectively). It has to be noted that leptin upregulation, typically observed in obese subjects, was independent from BMI, which was considered as a confounder. Likewise, transcription of three other genes was upregulated both in virus-positive and virus-negative specimens: trafficking regulator of GLUT4 1 (*TRARG1*; also called IFN-induced transmembrane domain-containing protein D3, FC = 3.11, FDR = 0.02 and FC = 2.64, FDR = 0.03, respectively); transmembrane 4 L six family member 19 (*TM4SF19*, FC = 4.25, FDR = 0.04 and FC = 3.03, FDR = 0.07, respectively); aquaporin 7B (*AQP7B*, FC = 1.57, FDR = 0.15 and FC = 1.7, FDR = 0.07, respectively).

## Discussion

**Fig. 8:**
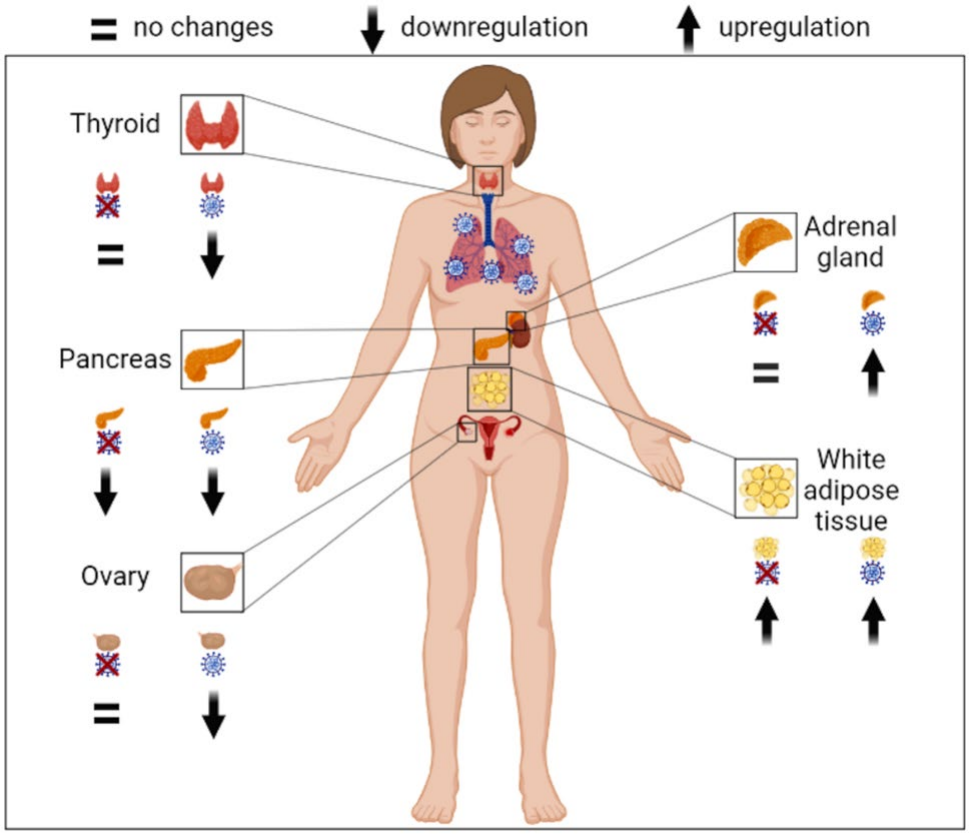
Summary of the study. Transcriptional deregulations in endocrine tissues from lethal COVID-19 cases are organ-specific. In thyroid, pancreas and ovary endocrine-specific genes are downregulated, while in adrenals and WAT, they are upregulated. Deregulations occur when SARS-CoV-2 directly invades thyroid, ovary, and adrenal gland; however, mRNA transcript changes in pancreas, and WAT are independent of virus infection of the organ (created with BioRender (https://biorender.com/))

COVID-19 is a pulmonary and systemic disease. Though the rates of hospitalization and death are decreasing due to vaccination, improvement of therapies, and the selection of less pathogenic SARS-CoV-2 variants [27], the involvement of extrapulmonary organs remains a long-term threat. Among post-acute sequelae, endocrine and metabolic disorders are relatively common [28]. Though a variety of studies reported alterations of circulating hormones and metabolites both in the acute phase and afterwards [20], transcriptional changes in endocrine organs have been barely investigated. Here, by comparing mRNA transcript levels of genes expressed in endocrine organs of COVID-19 cases vs. controls, two major findings emerge: (a) ISGs are consistently upregulated in virus-containing tissues of five different endocrine organs. Surprisingly, in WAT, the upregulation of ISGs occurred also in virus-negative tissue specimens, though less markedly; (b) the deregulated transcription of endocrine-specific genes is strictly organ-specific (Fig. 8).

Consistent with our previous results [4–6], activation of ISG genes is regularly observed in endocrine organs infected by SARS-CoV-2. It is known that the production of type I IFNs can be elicited in almost every cell type and that IFN receptors (IFNARs) are expressed on almost all cells, allowing them to acquire an antiviral state [29]. However, in WAT, IFN responses were activated even in the absence of virus within the tissue. Though the finding may seem counter-intuitive, IFN activation in non-infected cells may also be caused by non-viral stimuli such as xenogeneic or autologous nucleic acids through the STING pathway [30] and autocrine signaling turning on the IFN factor IRF7 [29]. In WAT of COVID-19 cases, it is of interest to note that leptin transcription was upregulated independently of the presence of virus in the tissue. Leptin, in addition to its hormonal effects, is known to elicit the production of pro-inflammatory cytokines in lymphoid cells [31]. Severe COVID-19 cases are characterized by high levels of circulating leptin [32]. Likewise, hyperleptinemia is common in obesity and associates with chronic immune activation [33]. Our results show that, even after adjusting for BMI, sex, and age, leptin transcript levels remained significantly higher in WAT of COVID-19 cases compared to controls.

Adrenal glands positive for SARS-CoV-2 showed a substantial upregulation of *HSD3B2*, *CYP17A1*, and *CYP11B1*. These genes encode for enzymes converting steroids to adrenal hormones. While the first two enzymes act in the synthesis of a wide range of steroids, *CYP11B1* is specifically involved in the conversion of progesterone to cortisol [34–36]. As a note, the upregulated transcription of the above genes does not seem to support primary adrenal insufficiency in COVID-19.

Apparently, a controversial scenario was detected in ovaries positive for SARS-CoV-2: downregulation of crystallin gamma D (*CRYGD*) and upregulation of the 5-hydroxytryptamine (serotonin) receptor 1A (*HTR1A*) gene. By sequence and structure, crystallin gamma D is similar to crystallin beta, but the latter is monomeric [37]. Currently, no clear functions have been attributed to gamma-crystallin in ovaries, but beta-crystallin does influence female fertility by regulating apoptosis in granulosa cells and follicular atresia [37]. Hence, the downregulation of *CRYGD* may be consistent with alterations of the menstrual cycle observed in women recovering from COVID-19 [25]. In addition, the enhanced expression of the serotonin receptor 1A is in line with decreased serotonin serum levels observed in severe COVID-19 cases [38]. Similar to what is found in humans, in mice, the Zika virus also targets the ovaries inducing a type I IFN response associated with disordered steroidogenesis [39].

In thyroid, alterations of gene transcription were only observed when SARS-CoV-2 was detected in the tissue. Changes affected factors associated with thyroid dysfunction [40]. Transcription of *ZNF804B* and *FOXE1* was downregulated; *ZNF804B* is possibly associated with antiviral defense [41], while *FOXE1* promotes the expression of multiple thyroid-specific genes encoding for thyroglobulin, thyroid peroxidase, thyroid dual oxidase 2, pendrin, and other transporters [42]. The *TSHR* gene was also downregulated. Since serum *TSH* levels are generally low in mild to severe forms of COVID-19 [43], low levels of the TSH receptor indicate a possible impairment of the HPT axis. In a scenario of suppressed function, the enhanced expression of thyroid peroxidase may appear unjustified. However, it is known that non-endocrine regulatory mechanisms may operate during viral stress responses [44]. Indeed, thyroid peroxidase is a major autoantigen in thyroid autoimmunity and a key player against oxidative stress [45].

Finally, the exocrine and endocrine pancreas deserves a separate comment. First, in COVID-19 cases, the pancreas is the only tissue for which alterations of gene transcription are seen in the absence of activated IFN responses and independently of the presence of the virus in the tissue. Second, the expression of pancreatic lipase-related protein 1 (*PNLIPRP1*) is enhanced. Differently from its paralogs (pancreatic triacylglycerol lipase and *PNLIPRP2*), *PNLIPRP1* lacks lipolytic activity and also inhibits pancreatic lipase [46]. Thus, enhancement of a lipase inhibitor may represent a defensive response under conditions of organ damage. Third, and more important, the results show that two beta cell genes coding for insulin (*INS*) and islet amyloid polypeptide (*IAPP*) are downregulated. Both hormones regulate circulating glucose levels, and their expression is downregulated also in diabetes. These findings support a possible dysfunction of beta cells in COVID-19 and remind that, in beta cells, the stress response associated with a reduction of intracellular proinsulin may activate inflammatory pathways [44, 47]. While in virus-positive cases, the suppressed expression of beta cell genes might be due to the direct viral infection, the determinant of such deregulation in virus-negative cases might be elusive. However, 75% of subjects of the COVID-19 cohort with virus-negative pancreatic tissues were treated with glucocorticoids, which are known to antagonize insulin signaling and production [48]. Unexpectedly, the transcription of somatostatin (*SST*) gene was downregulated only in virus-negative pancreas specimens. Somatostatin is produced by pancreatic delta cells and regulates the production of pituitary growth hormone, thyroid-stimulating hormone, and hormones of the gastrointestinal tract [49]. Contextualization of somatostatin downregulation in COVID-19 needs further analysis. Of note, glucagon mRNA levels were not altered in virus-positive nor in virus-negative COVID-19 cases compared to controls, thus suggesting normal alpha cell functioning.

This study has some limitations. First, the sample size per group in each investigated endocrine organ is relatively small, though more than a hundred tissues were analyzed. Second, the significance level was set at FDR = 0.25, a way to identify significant features in relatively small size groups. On the other hand, important sources of variation such as age, sex, and BMI have also been considered in the analyses. Third, SARS-CoV-2 viremia has not been evaluated in the course of the disease, and circulating glucose and hormone levels were not available. Fourth, since the investigated COVID-19 cases were collected in 2020 and 2021, the results refer only to the original Wuhan strain and the alpha variant of SARS-CoV-2, not to the currently predominant variants. Finally, only a few genes highly specific for each organ were evaluated. Nevertheless, the specificity and functional relevance of the investigated genes are allowed to recognize alterations of interest for translational medicine.

In conclusion, transcriptional alterations of endocrine genes in individuals who died because of COVID-19 are specific for each endocrine organ. In most cases, changes were observed only when the SARS-CoV-2 could be detected in tissue. While infected ovary and thyroid showed downregulation of tissue-specific genes, in adrenals and WAT, transcription of endocrine genes was enhanced, possibly as part of a stress response to infection. Notably, in beta cells, hormone genes were suppressed also in the absence of virus. This is reminiscent of type 1 diabetes where beta cell functions are inhibited in an inflammatory context [47]. Our findings provide evidence that endocrine dysfunction may arise in COVID-19, especially when the virus invades endocrine organs. Though vaccination and prior infection have a protective role against the acute and long-term effect of COVID-19, clinicians must be aware that endocrine manifestations can derive from virus-induced and/or stress-induced transcriptional changes of individual endocrine genes.

## Supplementary Information

Below is the link to the electronic supplementary material.

Supplementary file1 (XLSX 30 KB)

## Data Availability

The datasets generated during the current study are available from the corresponding author on reasonable request.
